# “You get to know the people and whether they’re talking sense or not”: Negotiating trust on health-related forums

**DOI:** 10.1016/j.socscimed.2016.06.029

**Published:** 2016-08

**Authors:** Ellen Brady, Julia Segar, Caroline Sanders

**Affiliations:** University of Manchester, United Kingdom

**Keywords:** United Kingdom, Internet forums, Online peer support, Trust, Health information

## Abstract

The internet is increasingly being used as a source of health advice and information by individuals with long term conditions (LTCs). Specifically, online forums allow people to interact with others with similar conditions. However, it is not clear how online health information is assessed by those with LTCs. This study aims to address this gap by exploring how individuals with contested and uncontested LTCs utilise internet forums. Semi-structured interviews were conducted with 20 participants with ME/CFS and 21 participants with type 1 and 2 diabetes and analysed using thematic analysis. Participants were recruited via online and offline routes, namely forums, email lists, newsletters, and face-to-face support groups. The findings indicate that the use of online forums was a complex and nuanced process and was influenced by a number of individual and illness-specific factors. Participants trusted those with similar experiences and perspectives as themselves, while also valuing conventional biomedical information and advice. By accessing support online, forum users were able to draw on a personalised form of support based on the lived experiences of their peers. However, the role of digital literacy in developing and maintaining online relationships must be acknowledged.

## Highlights

• The use of online forums by individuals with LTCs is a complex and nuanced process.
• Participants trust those with similar experiences and perspectives as themselves.
• Users can access a personalised form of support based on the experiences of peers.
• Role of digital literacy in developing online relationships must be acknowledged.

## 1 Introduction

Rapid advances in technology and internet use have led to an increasingly evolving body of research and practice in the area of eHealth. This paper addresses a specific subsection of this growth, namely the use of online forums to access health information by individuals with long term conditions (LTCs) and how peer-sourced online health advice and information is evaluated by forums users.

The most recent statistics from the UK suggest that nearly 70% of British internet users searched for health information online in 2013, while 33% contributed to an online forum (Dutton and Blank, 2013). Online forums function by enabling individuals to engage in supportive interactions with others facing similar experiences, challenges, or problems (Coulson et al., 2007). For health-related queries, the convenience of use enables individuals to receive informational and emotional support whenever they wish rather than waiting for a scheduled appointment (Elwell et al., 2011).

Hardey (1999) suggests that the array of information and advice available on the internet enables users to develop and redevelop their identity in a way that goes beyond the concept of a patient as a disembodied medicalised case. Individuals control the content and flow of the information available to them, rather than receiving information through a healthcare professional. This has been echoed by a number of researchers (e.g., Barker, 2008, Pitts, 2004) who suggest that the autonomous nature of internet use empowers individuals. Forums allow individuals to access a collective pool of health-related information and social support borne from shared experiences. By communicating online with others, forum members can build relationships and determine whether or not to trust fellow posters.

Consequently, it is important to emphasise that information seeking and sharing online extends beyond a mere channel of information. The internet offers information that users interact with, attaching and detaching meaning in relation to their daily health practices and everyday experiences (Kivits, 2006, Kivits, 2009). This is particularly worth noting in the context of the digital divide, where inequalities associated with education and income are linked to lower use of the internet (Cotten and Gupta, 2004). In recent years, focus has shifted to the importance of online literacy as a contributing factor to online health inequalities. Neter and Brainin (2012) suggest that the digital divide extends beyond connectivity, with marked differences in how individuals utilise and interpret the internet as a source of health information and advice. Illustrating this, Li et al. (2014) reported that those with high eHealth literacy were more likely to go online after a doctor’s visit and to look at more specialised health information.

While the use of the internet in healthcare has been a growing area of study, it has also prompted a number of reactions. Nettleton et al. (2005) suggest that these reactions can be broadly categorised into three responses. Firstly, the use of the internet for health information can be viewed in a ‘celebratory’ context, as a readjustment of a power imbalance between patients and health professionals. Secondly, responses can be classified as ‘concerned’, which, they report, is predominately the perspective of the medical profession. This reaction stems from a worry about the quality of health information available online and views lay people as having insufficient expertise to assess the reliability of information available online. Lastly, internet use can be viewed as contingent and embedded, whereby individuals make ‘reasonable’ assessments of online information in the context of their own health, illness, and offline lives. This approach highlights how the use of the internet for health information blends with other sources of help, advice, and support. Though this view is one that is supported by much of the current literature (Bell, 2014, Kivits, 2009), it nonetheless requires further scrutiny.

For complex or emergency situations, patients are likely to place high levels of trust in healthcare professionals, and may devolve decision making to those within the health care system (Fotaki et al., 2005, Meyer and Ward, 2013). By contrast, for LTCs, individuals are frequently armed with increased levels of information, along with their experiential knowledge. While trust in health care systems is not disregarded in chronic conditions, it is instead conditional and negotiated between patient and professional (Fotaki, 2014). Given this, it is necessary to explore how individuals with LTCs assess and trust the peer-sourced health information that they encounter online.

### 1.1 Assessing peer-sourced health information online

The level of information provided on forums increases the risks of misinformation and misperceptions being transmitted. This is something that has been acknowledged by forum users, with members of an online food allergy forum suggesting problems with the accuracy of information on the forum (Coulson and Knibb, 2007). Wang et al. (2008) found that degree of perceived similarity a receiver ascribes to a message source in knowledge, experience, background, and views was crucial in evaluating the information available in online forums, rather than the credibility of the information. This indicates that while online peer support and advice may be beneficial for those with LTCs, there is a risk that misinformation will be accepted due to the nature of transmission. Despite this, Esquivel et al. (2006) reported that a tiny minority of messages posted to a breast cancer mailing list contained statements that were found to be false or misleading. Likewise, Armstrong et al. (2012) found that no inaccurate information was shared on a forum for people with diabetes. When a controversial opinion was posted, it was soon negated by other forum users, suggesting that online forums can act as ‘self-policing’. In addition, forum moderators typically provide guidance around appropriate forms of information for each forum (Mudry and Strong, 2013).

Similar results have been reported across a range of studies. Sillence and Mo (2014) reported that forums for individuals with prostate cancer provided diverse information and advice, with a deferral to healthcare professionals and a detailed consideration of individuals’ own health and circumstances. Giles and Newbold (2011) described a high level of deference to medical expertise on mental health forums, even ones which had an explicitly anti-recovery focus, while van Berkel et al. (2015) report that forum users across a range of conditions were frequently directed to healthcare professionals by other posters.

It has been suggested that exposure to ‘experiential’ information online, e.g., *“I can’t tell you what to do but this is the decision I made and why”* (Sillence and Mo, 2014, p. 245), is highly valued by individuals (Rozmovits and Ziebland, 2004). Notably, prior research indicates that people draw on others’ experiences as part of their healthcare decision making, integrating them into their existing medical evidence base (Ziebland and Herxheimer, 2008). This is not to say that drawing on individual narratives in assessing health information online is an unsystematic process. Sillence and Mo (2014) suggest that the public nature of online forums motivates users to analyse information in a methodical manner and to present their thoughts in a considered and deliberate way. Posters providing advice tend to do so in line with the limits of their individual experiences and the boundaries set out by the member seeking advice (Sillence, 2010).

Armstrong et al. (2012) described a diabetes forum as a place where it was acceptable to express slightly unorthodox views. However, these views were invariably framed in the context of medical information or knowledge to indicate that forum members were knowledgeable and authoritative about their condition (Armstrong et al., 2012). In addition, the acknowledgement of these views as unorthodox allowed participants to draw the forum’s attention to the fact that these views may not be acceptable, providing the community with an opportunity to evaluate if the views were suitable for expression within the forum.

This suggests that notions of credibility and legitimacy online are not fixed and can evolve depending on the constitution of a group. As forum members establish legitimacy and authority, they become part of a community. As such, they influence and are influenced by group norms (Smithson et al., 2011). In turn, the community constructs the definition of acceptable discourse and reinforces the authority of individual writers (Galegher et al., 1998). In order to explore these notions in relation to LTCs, it is necessary to give in-depth consideration to the nature of forum usage by specific communities and to examine how trust is determined and negotiated. The communities and conditions selected for inclusion in this paper are outlined in the following section.

## 2 Method

Semi-structured qualitative interviews were conducted with UK-based individuals with LTCs from two populations who represented a number of illnesses and viewpoints. One sample consisted of individuals with type 1 and 2 diabetes. Diabetes affects more than 5% of the British population and has been highlighted by the NHS as a key focus of the effort to improve chronic disease management in the UK (Department of Health (2004)). The other sample consisted of individuals with myalgic encephalomyelitis/chronic fatigue syndrome (ME/CFS). It has also been suggested that online forums may offer a particular role for individuals with ‘contested’ chronic illnesses due to the lack of agreement surrounding the nature and treatment of the condition (Chen, 2012). Given that ME/CFS is a contested illness, characterised by the absence of a biomedical explanation (Schoofs et al., 2004), many people struggle to receive a diagnosis and support from health professionals (Huibers and Wessely, 2006). It was therefore anticipated that examining the use of internet forums by individuals with diabetes and ME/CFS would provide a contrasting and illuminating context in which to explore how forum users assessed health-related information online.

### 2.1 Interviews

A qualitative approach was selected as it allowed for an exploration of the perspectives of individuals with LTCs. Semi-structured interviews enabled participants to discuss the topics that were relevant to their health and use of internet forums. A broad interview schedule, covering the participant’s health and illness, informal and formal support networks, and their use of the internet and internet forums, was developed for use with all participants. It should be emphasised that the schedule provided a guide for the interview rather than a prescriptive itinerary. Interviewees were given space to express their own opinions and ideas, and, in many cases, their responses shaped the order and structure of the interview (Dyer, 2006).

### 2.2 Participants

41 participants completed interviews, 20 with ME/CFS, 12 with type 1 diabetes, and 9 with type 2 diabetes. To ensure that a range of perspectives were considered, interviewees were not required to have any previous experience with internet forums and recruitment took place both online and offline, via internet forums, face-to-face support groups, email lists, and research networks. Interviewees were drawn from across the UK and the majority of the respondents were female (*n* = 28). The mean age was 50 (age range = 18–82 years), reflecting the broad range of experiences as well as the age profile of the conditions.

Participants were offered the option of face-to-face or phone interviews with the first author; many (*n* = 29) chose to participate by phone. Interviews ranged in length between 20 min and two hours, with most lasting in excess of 45 min. All interviewees described themselves as white. Notably, the majority of participants (71%, *n* = 29) had completed at least a higher education degree or equivalent. The latest census data suggested that in 2011, just 27% of the population of England and Wales had received a degree or higher (ONS, 2014), indicating that participants in the present study are educated to a higher level than the general British population.

### 2.3 Data analysis

Interviews were recorded and transcribed verbatim. The anonymised interview transcripts were imported into a qualitative data analysis computer software package, ATLAS.ti version 7, in order to carry out the analysis. It should be noted that the use of a software package merely provided a tool to organise and review the data during the analysis process, rather than offering an objective method of analysis (Mauthner and Doucet, 1998). Each transcript was read through several times, and notes were made in order to make note of preliminary connections between interviewees.

A thematic method of analysis was employed, with a view to examining comparisons and contrasts across participants and within cases. Thematic analysis was chosen as it provided a flexible approach to analysing qualitative data and involves identifying themes in a body of data (Braun and Clarke, 2006). Themes were considered to capture something important about the data and to represent a level of patterned response or meaning within a data set. This process allows the development of a conceptual scheme which enables further interrogation of the data (Basit, 2003).

The analysis followed an iterative process. A coding frame was devised by the first author comprising the initial themes identified within the data. Following this, the data were coded according to these themes. Initially, these codes were broadly descriptive, and related directly to the content of interviewees’ transcripts, rather than any more subtle nuances within the data. For example, references to an interviewee’s family were coded as ‘Family’; and so on. As coding continued, categories were further refined into sub-categories or aggregated to form higher level categories, as the initial coding frame did not sufficiently capture the complexities of the data. The coding frame was continually revised and transcripts were reviewed on an ongoing basis by all authors to ensure that additional codes were applied.

### 2.4 Ethics

Ethical approval was granted by the University of Manchester research ethics committee. Any identifying information was removed from the interview transcripts and all participants have been assigned pseudonyms. Each participant was provided with an information sheet and encouraged to contact the researcher with any questions both before and after the interview. Signed consent was received from all participants; for telephone interviews, the consent form was posted in advance along with a stamped addressed envelope to return the signed form.

## 3 Results

By communicating online with others, forum members developed relationships and gradually determined whether or not to trust fellow posters. In this way, trust was negotiated between peers. This section describes the nuanced development of trust that took place on health-related forums. Pseudonyms have been used for all participants.

### 3.1 Birds of a feather: collective identity and individual markers of trust

In establishing trust on internet forums, the relationship between users was pivotal. Participants valued receiving information from forum members who had a similar perspective to themselves. Previous research indicates that internet users are more likely to assess user-generated information as credible when they perceive the user as similar to themselves (Flanagin et al., 2013). This was illustrated by James, who described how the people that he gravitated towards on a forum for individuals with ME/CFS were those who held similar values. While he found the forum to be a useful venue for keeping up to date with research and developments around ME/CFS, he felt that a number of members were overly concerned with challenging what they perceived to be a psychological rather than biomedical approach in ME/CFS research. By contrast, the people with whom he had developed a friendship took a more pragmatic approach, and were content to dismiss research that they felt promoted a perceived psychological agenda, *“They know that they’re not suffering from any kind of depression or mental illness or whatever and therefore it really doesn’t matter what study you do and what link you show”* (James, ME/CFS, 51–55 years). As a result of this shared perspective, James grew to respect certain members of the forum and to value their opinions. Similar to *“real life”* processes of friendship, he had built up trusted support networks with certain members based on their shared perspectives. This also suggests that ‘value homophily’ plays a role in establishing trust on online forms, where users gravitate towards those with similar attitudes, beliefs and behaviours (Wang et al., 2008).

This was echoed by Laura, who described how forum members’ backgrounds were crucial in evaluating their advice and assessing how it applied to her condition. As she participated in a lot of sport, many of her queries about her type 1 diabetes related to the impact of exercise on diabetes and insulin dosage. Consequently, she was more likely to trust the advice of someone who she felt led a similarly active lifestyle, *“The person that gave me this advice, I knew they were an athlete anyway and had a lot of exercise with diabetes, so I would take that advice and use it”*. Equally, forum members who had similar physical needs as Laura were valued, *“If somebody you know is very, for example, very short, petite on a very low dosage of insulin throughout the day … I’m quite sporty so I’m, sort of, chunky but not overweight, you know, what would work for that person wouldn’t probably work for me”* (Laura, type 1 diabetes, 31–35 years). Accessing information of this nature allowed her to take a more personalised approach to her own health care than could be provided within a formal healthcare setting (Keeling et al., 2013).

The relationships between forum members developed over time and through a number of interactions. While members who had been actively participating in forums over a number of years were valued, it was clear that it was not just their length of service that was a defining characteristic in evaluating these members. Consistently providing sensible and trustworthy advice led to members becoming valued and trusted within a forum, “*You get to know the people and whether they’re talking sense or not … you’re more likely to take the word of somebody who’s respected than somebody who walked in ten minutes ago”* (June, type 1 diabetes, 66–70 years).

### 3.2 Gaining credibility points: constructing a knowledgeable identity online

There were a number of ways that forum members could establish their credibility as both information providers and patients. Appearing to be educated or articulate meant that an individual’s advice or information was typically considered to be trustworthy, *“If it comes across as reasonable and educational, you know, you kind of trust in it, rather than someone who types ‘lyk dis’”* (Jessica, type 1 diabetes, 18–25 years). However, formal qualifications were not explicitly required in order for an individual to become established as credible. Instead, forum users could gain the trust of others by providing information about their condition. For some interviewees, a diagnosis of their condition confirmed by a healthcare professional was important, as opposed to a self-diagnosis. While this may seem counterintuitive, particularly considering the ambivalent relationship between individuals with ME/CFS and the medical profession, this echoes similar research around online mental health communities which embody a resistance to the medical community (Giles and Newbold, 2011). This suggests that, even within communities which aim to challenge accepted medical discourse, external markers of legitimacy are valued.

For example, Nicole spoke about her frustration on reading advice and information from a fellow ‘patient’ with ME/CFS online over a series of months, only to learn later on that the forum member had not received a diagnosis, despite repeated contact with medical professionals. As a result of this experience, she was inclined to place more value on forum users who had received a diagnosis, and was more likely to trust their advice.

*On [name of forum], you have to say whether you’re a sufferer, whether you’ve self-diagnosed, whether your GP has, so you have an idea of some people who, you can see where they are and how long they’ve been suffering from, as to, whether or not to take their advice.* (Nicole, ME/CFS, 26–30 years).

The length of time since diagnosis was also seen as evidence of a forum member’s credibility as a source of advice and information online. This was particularly true for those with diabetes, as the length of time was indicative of more experience with the condition, *“I’ve tried some things that they’ve suggested because I think from what they’ve told me, they have had more experience. So I’m going on the fact that they have had longer experience, longer time”* (Emma, type 1 diabetes, 41–45 years), but was also seen as a sign of a healthy lifestyle, *“And there are lots of people on the forum who have had it for sort of like 20 years and they still haven’t got any of these terrible things, so that’s good”* (Patricia, type 2 diabetes, 66–70 years).

Having experience of a particular procedure or treatment meant that an individual was likely to be viewed as a trusted source of information by members. For example, Patricia described her experiences of seeking advice in advance of her first retinal screening after her diagnosis with type 2 diabetes. When she spoke to her local surgery, she received reassurance, but not a very detailed response, *“I asked the people at the surgery and they just said, oh, it’s absolutely routine, you just go to the hospital, they’ll put some drops in your eyes, take some pictures and that’s it”*. By contrast, forum users who had experienced the procedure were able to provide her with answers to her specific questions, *“I thought, OK, I’d like to know a bit more about this, can I drive, that kind of thing, and I found the forum people were very helpful”*. Though she received a range of responses and some diverging opinions, she considered the responses from those who had experienced the procedure to be the most credible sources of information. The ‘lived experiences’ of these forum members meant that their views provided her with a source of support that could not be accessed through traditional medical sources of information.

Forum members’ opinions and perspectives were not just accepted uncritically, however. Sharing detailed information about one’s experiences meant that these experiences could be assessed and evaluated in relation to other people’s conditions and lifestyles. This was illustrated by Ian, who described how knowing about forum members’ backgrounds, such as their condition, length of time since diagnosis, and typical blood sugar control, influenced his evaluation of their experiences. He used this detailed information to assess how credible these individuals were as information providers and to determine if he would utilise their experiences to improve how he managed his own diabetes.

In addition, participants reported that they valued advice and information from forum members who had previously suggested something that had been successful or helpful for them. Interviewees referred to trusting those who provided advice that they had successfully utilised, *“You do value everyone’s opinion and advice but others are probably more valuable to you than others because they might have suggested something that works for you. And obviously with that, then you gain a natural trust from someone from that”* (Daniel, type 1 diabetes, 26–30 years), and giving them ‘credibility points’.

Interviewees also drew on the experiences of other forum members in assessing the information provided by an individual poster. Mark referred to the *“feed of information”* and *“pool of collective experience”* that forums provided, allowing members to assess the information that they were receiving against the benchmark of other information and experiences that had been shared on the forum. Comparing responses to the responses from other members enabled posters to assess the information across a spectrum of knowledge, rather than regarding it in isolation, *“If four or five people are saying a similar thing then you think, well, OK, that’s an interesting idea, but the sharp end is you might give it a go”* (Mark, type 1 diabetes, 41–45 years). Forum members could therefore access a group consensus within a single medium, which allowed them to easily distinguish which sources of advice and information were credible. This in turn allowed them to select information which they could apply to their own lifestyle in order to improve their health and condition management.

As a result of this, forums appeared to be somewhat self-policing. Forum members were able to draw on various pieces of evidence to establish themselves as credible sources of health advice and information. As suggested by Metzger et al. (2010), posters established a ‘bottom-up’ assessment, where the quality of information could be assessed and constructed via the online community. Members were able to evaluate information in line with their own experiences and knowledge base, as well as drawing on the collective knowledge on the forum. Users were frequently reminded by forum moderators that members were not medical professionals and that they should seek professional advice if required, *“I mean, we always end up with, of course, they can’t give medical advice, you should, you know, if you want to do this, go and discuss it with your doctor sort of thing”* (June, type 1 diabetes, 66–70 years). Consequently the information presented on forums was typically framed in the context of experiential information rather than directive medical advice and often involved a deferral to healthcare professionals (Sillence and Mo, 2014).

### 3.3 Establishing evidence-based lay knowledge

A number of participants expressed concerns about the accuracy of information accessible on the internet. This was particularly pertinent for those with ME/CFS, many of whom reported that they had encountered misinformation online. Much of this misinformation built upon the lack of clarity around the condition and was targeted at potentially vulnerable individuals who were unable to access support through traditional sources. Karen described how she had encountered many websites after her diagnosis which touted cures for ME/CFS, usually for commercial gain. She was concerned that these purported cures could not only negatively affect individuals financially and physically, but could also instil them with a false sense of hope and optimism.

*There were a lot of websites saying that they could cure it and obviously I knew myself that there’s no cure … If you invested money and time into something that wasn’t going to work anyway you’re going to make yourself, it would do harm, it affects you emotionally, financially and probably physically, and mentally as well.* (Karen, ME/CFS, 41–45 years).

Though many participants expressed concern about the veracity of information online, their concerns predominately centred on the possibility that users other than themselves may be susceptible to this misinformation. Given that the majority of interviewees were educated to degree level or higher, many interviewees drew on their own education or employment background in order to evaluate the information that they encountered on the internet, *“I do have a few years of my working background working in evidence-based medicine, so I know how to read a study report and understand its shortcomings and its benefits”* (Julie, type 2 diabetes, 46–50 years).

There was a perception among interviewees that they themselves had the educational background, *“Because having done a BA, you know, I’m perfectly good at researching online, you know, I do a lot of research and I find what I need to find and use it appropriately”* (Carol, ME/CFS, 66–70 years), research skills, *“I use my own scientific background, to determine what I’m reading is accurate, which perhaps puts me in a different situation from people who haven’t a long lasting scientific background”* (William, type 2 diabetes, 76 + years), or personal knowledge, *“I like to think I’ve got the knowledge to be able to figure out what’s the good advice, what’s the bad advice, what’s going to work for me, what’s not”* (Daniel, type 1 diabetes, 26–30 years), to discern good information from bad information online, but that others may not be able to do so quite so easily. This suggests that while individuals were aware of potential risks about health misinformation online, they believed that their own ability to appraise and evaluate information overrode these risks.

However, it is necessary to contextualise this finding within the existing literature. As highlighted previously, the educational background of participants was likely to have influenced their use of internet forums. Highly educated individuals have higher levels of online literacy and are more likely to find information online that improves their health (Dutton and Blank, 2013, Neter and Brainin, 2012). As a result, the self-reported abilities of forum users to identify misleading or dangerous information online are shaped by factors such as education, socioeconomic class, or social capital (Bell, 2014).

## 4 Discussion

This paper explores how trust was developed and negotiated on health-related forums. Participants reported that they were attracted to members who in line with their own beliefs and experiences. Markers of trust online appear to be situated within individuals’ everyday lives, and as a result, forum users’ assessments of information sources online are contingent and embedded (Nettleton et al. 2005). Reflecting on this shows support for Kivits’s (Kivits, 2006, Kivits, 2009) argument that the internet goes beyond an information source; instead, it provides information that individuals interact with, depending on their daily lives and health practices. The development of trust online was a nuanced process, with notions of credibility varying between forum members and are contextualised by an individual’s own background.

There were suggestions that participants’ use of internet forums was framed by dominant biomedical discourses. Despite the divergent perspectives of healthcare professionals and patients around the nature and treatment of ME/CFS (Salmon et al., 2007), within the present study there was a broad deference to conventional medical practices around the condition. For example, on ME/CFS forums, a formal diagnosis of ME/CFS represented an external marker of an individual’s credibility and therefore legitimised a poster as a source of advice and information. Likewise, Whelan (2007) reported that a diagnosis of endometriosis represented a key turning point for individuals. It signalled their entry into the patient-centred community of individuals with endometriosis, which, despite the difficulties that many experienced in receiving a diagnosis, was policed by the discursive act of medical labelling. The echoes of this in interviewees’ accounts indicates that forum members situated the advice and information shared online in the context of their biomedical knowledge about their health and illness (Sillence and Mo, 2014).

The findings also highlight the benefit of developing relationships online. Interviewees became aware of forum members with similar backgrounds or perspectives to themselves due to interactions that occurred over time. While research suggests that those who lurk on forums can receive similar benefits to those who are active posters (Mo and Coulson, 2010, van Uden-Kraan et al., 2008), the present study shows that interacting with others online allows forum members to develop a pool of peers whom they consider to be useful and trusted sources of information. The degree of familiarity between forum members meant that individuals were able to access a personalised form of support which was targeted to their specific lifestyle and needs, compared to that provided by healthcare professionals (Keeling et al., 2013).

This argument is echoed in other studies. There have been suggestions that, with continued participation in a forum, the relevance of the narratives, advice, and information available online increases. Individuals can build up a base of knowledge about their condition, comprisingnew information as well as experiences that enforce the reliability of the information and add credibility to different sources (Johnston et al., 2013). As individuals become connected to communities, the information provided through these networks becomes more meaningful and accessible to the participant (Johnston et al., 2013). This research builds on this notion to highlight how these connections are instrumental in individuals with LTCs assessing and evaluating the information that they encounter via online forums.

Developing relationships with others online enabled forum members to piece together a detailed picture of posters’ individual circumstances and to look for particular indicators of credibility. Forum users typically responded to requests for personal experiences in a narrative form, providing readers with enough information to assess how applicable the advice is to their own situation (Sillence, 2013). Within the present study, interviewees could draw on their own knowledge and experiences as both a patient and a forum user to assess the advice and information that was being provided to them. This allowed forums to be broadly self-policing (Armstrong et al., 2012), as forum members were able to access a group consensus within a single medium. In this way, trust was constructed on the forum, with the community assessing pieces or providers of information as credible.

Considering that the aim of this research was to explore the experiences of individuals with contested and uncontested LTCs, it is worth reflecting on the implication of this finding in this context. While participants reported using a number of strategies to assess the information that they encountered on the internet and suggested that they took a critical approach towards looking for health information online, many of these specific strategies can only be attributed to individuals with diabetes. While individuals with diabetes were typically required to seek advice and information online on an ongoing basis and to utilise it into the daily management of their condition, the lack of consensus around the treatment for ME/CFS meant that the same procedures did not apply. In addition, the absence of biomedical markers of progress such as weight loss, stabilisation of blood sugar levels, and reductions in medication meant that forum users with ME/CFS were often unable to draw on the same evidence base to assess the credibility of information provided on online forums. Individuals with ME/CFS may be prevented develop a network of trusted peers online, which might result in them experiencing difficulties accessing some of the reported benefits of forums (Coulson et al., 2007).

Lastly, the level of education of interviewees was frequently cited, both explicitly and implicitly, as a reason for their ability to safely navigate information online. Aspects of presentation online, such as literacy and logic, were highly valued in other forum members, and led to them being assessed as credible sources of information by interviewees. As a result, it is important to acknowledge the link between digital literacy and forum usage. Forum users who were literate online were able to successfully navigate online discussion groups and were seen trusted and valued members of the community. While the present study did not collect sufficient socioeconomic or other data to draw conclusions around health literacy, the findings indicate that guidance around navigating health information online may be particularly necessary for those with lower levels of online literacy (Diviani et al. 2015).

### 4.1 Implications of findings

Reading about the experiences of their peers online enabled individuals to receive advice and information about managing the day-to-day problems of living with an LTC. There were aspects of this support that were unique to online support and could not be provided by healthcare professionals, like the personalised care offered via forums members (Keeling et al., 2013). However, this support ‘bridged the gaps’ between traditional service providers and did not replace or supersede any of the existing supports or services available to those with LTCs. The support provided via online forums further assisted forum users in the day-to-day management of their condition through the lived experiences of other posters. This mirrored the role played by an individual’s family and friends in filling the gaps created by healthcare services by aiding with the practical and emotional challenges of living with an LTC (Piette, 2010).

Being logical and articulate were highly valued in other forum members and led to them being assessed as credible sources of information by interviewees. Considering the role that relationships played in individuals’ forum usage, where interacting with other forum members over a prolonged period of time led to posters receiving personalised help and support (Johnston et al., 2013), this indicates that those who do not conform to these standards may be excluded from developing these types of relationships. This highlights that the digital divide extends beyond the notion of barriers to access to barriers to literacy (Diviani et al., 2015), with those who are able to navigate both the social and technical aspects of forums (Marwick and Boyd, 2014) gaining the most benefit. Considering this, it is likely that an increasing reliance on electronic communication may merely help those who are already catered for within existing healthcare systems (Viswanath and Kreuter, 2007). In addition, more research is needed to explore the links between digital literacy and the development of trust on health-related forums.

As a result, a key area for further research is a sociological focus on issues relevant to inequalities in internet use. Research suggests that, with the right approach such as increased availability and appropriate training, online access may be a means of reducing inequalities associated with health and healthcare provision (Brodie et al., 2000, Connolly and Crosby, 2014, Cotten and Gupta, 2004). Thus care needs to be taken to ensure that the role of the digital divide and its impact is carefully considered in commissioning, conducting, and evaluating health services research.

## 5 Conclusion

Rather than making a binary decision to act or not to act on the information that they accessed online, forum users had a more nuanced approach to trust. The process of establishing trust online was embedded and contextualised within an individual’s beliefs about health and illness (Nettleton et al. 2005), their backgrounds and everyday lives (Kivits, 2006, Kivits, 2009), and their relationships with forum members (Johnston et al., 2013). In addition to the individual methods of navigating forums, trust was also constructed on forums. The shared experiences presented online enabled the community to collectively assess pieces or providers of information as credible.

In summary, this paper has highlighted and emphasised the contextual and nuanced role that forums play in the lives of individuals with LTCs. The process of accessing and utilising online support and health-related knowledge via forums was one that was embedded in users’ daily lives and healthcare practices. Rather than this process being a cause for ‘celebration’ or ‘concern’ (Nettleton et al. 2005), it was instead contingent on a number of individual, contextual, societal, and community-related factors.
